# Experiences with community engagement and informed consent in a genetic cohort study of severe childhood diseases in Kenya

**DOI:** 10.1186/1472-6939-11-13

**Published:** 2010-07-15

**Authors:** Vicki M Marsh, Dorcas M Kamuya, Albert M Mlamba, Thomas N Williams, Sassy S Molyneux

**Affiliations:** 1 Kenya Medical Research Institute (KEMRI) Wellcome Trust Research Programme, PO Box 230, Kilifi 80108, Kenya; 2 Centre for Clinical Vaccinology and Tropical Medicine, Nuffield Department of Medicine, University of Oxford, Old Road Campus, Headington OX3 7LJ, Oxford, UK; 3 INDEPTH Network, PO Box KD213, Kanda, Accra, Ghana

## Abstract

**Background:**

The potential contribution of community engagement to addressing ethical challenges for international biomedical research is well described, but there is relatively little documented experience of community engagement to inform its development in practice. This paper draws on experiences around community engagement and informed consent during a genetic cohort study in Kenya to contribute to understanding the strengths and challenges of community engagement in supporting ethical research practice, focusing on issues of communication, the role of field workers in 'doing ethics' on the ground and the challenges of community consultation.

**Methods:**

The findings are based on action research methods, including analysis of community engagement documentation and the observations of the authors closely involved in their development and implementation. Qualitative and quantitative content analysis has been used for documentation of staff meetings and trainings, a meeting with 24 community leaders, and 40 large public and 70 small community group meetings. Meeting minutes from a purposive sample of six community representative groups have been analysed using a thematic framework approach.

**Results:**

Field workers described challenges around misunderstandings about research, perceived pressure for recruitment and challenges in explaining the study. During consultation, leaders expressed support for the study and screening for sickle cell disease. In community meetings, there was a common interpretation of research as medical care. Concerns centred on unfamiliar procedures. After explanations of study procedures to leaders and community members, few questions were asked about export of samples or the archiving of samples for future research.

**Conclusions:**

Community engagement enabled researchers to take account of staff and community opinions and issues during the study and adapt messages and methods to address emerging ethical challenges. Field workers conducting informed consent faced complex issues and their understanding, attitudes and communication skills were key influences on ethical practice. Community consultation was a challenging concept to put into practice, illustrating the complexity of assessing information needs and levels of deliberation that are appropriate to a given study.

## Background

There is debate in the biomedical research literature over the exact nature of community engagement [1-3] but the overall goals are agreed to draw largely on ethical concerns, including demonstrating respect for, protecting and empowering participant communities and individuals and strengthening research processes [4-7]. While community engagement may not play a role in all types and settings for research [7], the potential importance of involving communities is particularly recognised in international health research [8-11]. The goals, and therefore forms, of community engagement have been described as varying in relation to power-sharing, for example from information dissemination to full partnerships [1]. Generally consultation is seen to represent a 'middle level' power sharing arrangement, through generating understandings that can be taken into account in research planning. For example, Dickert describes that consultation 'recognises and accommodates the relevant particularities of a given community for a specific project' [12]. Relevant to balance of power debates, others highlight the value of external Institutional Review Boards referencing documented community consultation to strengthen protocol review [13,14].

At the Kenya Medical Research Institute (KEMRI)-Wellcome Trust Research Programme in Kilifi, Kenya, community engagement activities draw on action research processes, congruent with Reason and Bradbury's (2006) description of "seeking to bring together action and reflection, theory and practice, in participation with others, in the pursuit of practical solutions to issues" [15]. In this paper we present data collected and analysed as part of this action research, approved by institutional and national science and ethical review committees (KEMRI Scientific Steering Committee and National Ethical Review Committee). The study includes systematic documentation of community meetings and staff training activities undertaken as part of a longitudinal genetic cohort study, the Kilifi Genetic Birth Cohort (KGBC). KGBC was designed to investigate the relationships between human genetic factors and the risks of severe childhood illnesses, including malaria and bacterial infections. KGBC will also contribute data to the MalariaGEN Consortium, comprising collaborators from 20 developing and developed countries to systematically study the genetic basis of resistance to malaria [16]. The proposal for KGBC was approved by the National Ethics Review Committee in Kenya and OxTREC, the Oxford Tropical Network Research Ethics Committee in the UK.

Research addressing population-level genetic susceptibility or resistance to disease (genome-wide association studies or GWAS) has attracted broad attention in the scientific and public media based on its potential to increase understanding of disease mechanisms, improve diagnostic techniques and provide targets for the development of new vaccines and drugs [17,18]. Amongst the ethical issues that have been debated for this relatively new and complex technology, many questions surround the ways in which informed consent processes can be supported, or even whether valid informed consent in its traditional sense can be achieved [19-23]. These difficulties are common to all countries, but are often more extreme in developing country settings [4,9,10,24]. This is emphasized by principles of distributive justice that suggest a need to ensure access to the potential health benefits of GWAS in countries that experience the highest global disease burden [25-27]. Difficulties in obtaining informed consent for GWAS have included communicating about technical but specific issues, such as inheritance and genes, and the relationships of these with health and illness. They also include the seemingly intractable problem of communicating about nonspecific processes, since consent in GWAS is typically sought for samples and data to be stored for future unspecified use. Differences in understanding may manifest themselves as therapeutic 'misconceptions' of research [24,28,29]. The role of relationships between research staff and participants is acknowledged as a key component to the communication process [30-34] so that the perspectives of both parties are important to address. Where samples are to be archived with future open access, recognized as critical to maximizing utility of the research endeavour [35], there are further issues of governance in relation to protection of the rights and interests of participants [26,36-41].

To date, there is limited documented experience of community perspectives on GWAS in developing countries, particularly in Africa, and the gap in empirical research on the ethical, legal and social implications of this approach has been recognized [26,27]. One aim of the MalariaGEN consortium is to address ethical aspects of GWAS in international research and studies are in progress in Kilifi to assess social and ethical implications of health research in this setting. Against this background, this paper aims to draw on documentation from action research around community engagement to explore the perceptions, experiences and concerns of community members, community leaders and field workers related to KGBC. The analysis of these findings aims to contribute to understanding the strengths and challenges of community engagement in supporting ethical research practice, focusing on issues of communicating about GWAS, the role of field workers in 'doing ethics' on the ground and the challenges of community consultation.

### The research setting and community

The KEMRI Wellcome Trust Programme (KWTP) http://www.kemri-wellcome.org is an international multidisciplinary biomedical research programme started in 1989 as a collaboration between the Kenya Medical Research Institute (KEMRI) and the Wellcome Trust, UK. The centre in Kilifi is situated in the District General Hospital (KDH) of this relatively poor, rural district. The community referred to comprises the geographic population of approximately 250,000 local residents who access health services at KDH and has been described elsewhere in more detail [42]. The majority are subsistence farmers belonging to the Mijikenda ethnic group, with less than 20% migration from other parts of Kenya. Local tourism, petty trading and employment in nearby larger towns provide cash income. Local administration is the responsibility of chiefs, working through assistant chiefs and village elders. Chiefs are civil servants with at least 12 years of schooling, drawn from the ethnic community they serve. They are seen by community members as essential gatekeepers for community activities, but not necessarily as their representatives [28]. The research centre is the largest employer in Kilifi, with over 600 employees and around 35 early and mid career scientists from East Africa. Around 7,000 participants are recruited into different studies each year, many of which draw on the Kilifi Epidemiological and Demographic Surveillance System (Kilifi Epi-DSS) [43] as an epidemiological tool. The centre works in close collaboration with KDH, and ensures that a consistently high standard of treatment is available to all inpatients in many departments, including the children's general and intensive care wards, regardless of their participation in research. Community engagement at the centre is supported by a centralised group of community facilitators [11] and draws on action research principles of continuous evaluation and adaptation. Currently, activities include regular discussions and workshops with community leaders, representatives (KEMRI Community Representatives, or KCRs) and other health stakeholders and support to research staff working at the interface between the centre and the community.

KGBC aims to recruit 12,000 infants between the ages of 3 and 12 months into a cohort that will be followed prospectively for the development of severe diseases or death, documented through two linked on-going surveillance systems for in patient records at the district hospital and the Kilifi Epi-DSS. With approval by local, national and international scientific and ethical review bodies, the study involves the collection of standardised data on disease risk factors and a 0.2ml blood sample for genetic analysis. Capillary blood samples are removed from the heel to ensure an adequate volume is drawn rapidly and with minimum discomfort. This heel was selected over the more common finger-prick site as the latter would not reliably provide an adequate volume and venous sampling was considered excessively invasive and technically demanding in small children. Mijikenda research staff (field workers), fluent in local and national languages and with secondary school education, visit families to conduct the informed consent process and collect information and blood samples according to the study protocol. Samples are screened in Kilifi for sickle cell disorder (SCD) as part of research on the health effects of the sickle cell gene (HbS) [44,45]. Where children are found to have SCD, results are returned within three weeks of testing, and parents are invited to attend a dedicated SCD clinic for confirmatory testing and family counselling, with transport costs provided. Where tests indicate a normal HbS gene or carrier status, families are informed of a negative result. The SCD clinic is run by KWTP and KDH to support children and families with SCD whether or not they are participants in KGBC or any other research. Staff at the clinic monitor children's progress, provide advice to parents on management, treatment for complications and other illnesses and offer routine prophylaxis and supplements (proguanil hydrochloride, folic acid and penicillin tablets) free of charge. On completion of KGBC, de-linked DNA samples and associated clinical and phenotypic data will be sent to the Sanger Institute, Cambridge, UK, for further genomic analysis.

## Methods

The qualitative research described in this paper was conducted as part of wider ongoing action research activities supporting community engagement across the centre. The methods used have included two main steps:

### 1. Document analysis

Reports generated by the Community Liaison group at KWTP during the planning, conduct and monitoring of project specific community engagement have provided the primary data for analysis. Given the action research nature of these activities, at least one note taker experienced in qualitative data collection attended all meetings to record issues discussed by hand and make structured observations of relevant contextual findings, for example, attitudes and emotions of participants, delays in starting meetings and adverse physical environments. The documents analysed included reports from:

i) 40 public meetings including a total of approximately 8000 local residents between October 2006 and March 2007

ii) 32 small group meetings including 255 religious leaders (80% Christian; 20% Muslim) in total from May to November 2007

iii) 38 small group meetings including 392 village elders in total between June and August 2007

iv) 30 meetings of KCR groups, including 5 meetings for each of 6 groups between 2006 and 2007. These 6 groups were purposively selected from a total of 14 to maximize diversity by representing different geographic areas (rural, periurban and urban) as well as groups with different meeting attendance rates (low or high).

v) 2 training workshops with KGBC field workers

Analysis of reports from KCR meetings was undertaken as part of a wider study on KCR roles, challenges and benefits by DK and SM as part of an MPH thesis. Analysis was based on a combination of inductive and deductive approaches; all text was closely read and coded line by line, with progressive categorization generated both from inductive codes and from the initial objectives of the study (qualitative content analysis) [46]. All other meeting and workshop reports were analysed by content to generate lists of the questions or issues raised and how these were responded to, alongside counts of their frequency across groups and, where possible, their predominance within groups (content analysis) [47]. This analysis was undertaken by two authors (VM and DK).

### 2. Discussions within community liaison group

The findings of the analysis from step one were presented to and discussed by all the authors and the wider community liaison group, who have been closely involved in the activities reported on this paper from the outset. These discussions have allowed triangulation between different perspectives on the same activities and identified some additional issues that are not explicitly raised in written reports. The latter contribute to findings for KGBC field workers and are drawn from the experiences of AM in managing field worker support supervision and VM and SM in facilitating the redevelopment of informed consent forms for national ethical review (see below).

## Results

### a) Interactions with field workers

Training and support supervision for KGBC field workers was planned carefully from the outset, given their key role in informed consent. These activities were jointly conducted by the KGBC research team and members of the Community Liaison group. In addition to training on the study itself, field workers attended a one week participatory workshop addressing communication skills and participants' rights in research, building on studies conducted previously in this community on local perceptions of research [28,48]. During training it became clear that, despite having worked for KWTP for some time, several field workers had a low understanding of the nature of health research, often conflating treatment and research activities at the centre, and demonstrating the same therapeutic 'misconceptions' of research that were commonly described by community members. Of particular importance, the wider context of national and international research review processes and the existence of national and internationally agreed research ethics principles were generally unknown. Instead, field workers discussed their work of recruitment in the context of a very challenging aspect of a job for which they were employed by KWTP. The scientific basis for KGBC was also difficult to comprehend, exacerbated by the conceptual gap between the research procedures experienced (a single blood sampling) and the putative social value (future malaria drugs and vaccines). Strategies were developed to address these issues during the training workshop, including participatory methods such as small group discussions and role plays. Following the initial training, one of the authors (AM) met with the fieldworkers on a weekly basis to discuss their experiences of recruitment, assess the community's response to the study and facilitate problem solving.

From the weekly meetings, a particularly important issue that emerged over time concerned the high degree of stress experienced by field workers in relation to recruitment, given the difficulties in communicating about the study. They had participated in a lengthy participatory training with experienced facilitators to acquire understanding of research and KGBC themselves, but now felt limited in their own skills and the time they should take in explaining these to potential participants. In part this occurred as a response to recognising community needs, that is, for busy mothers not to spend time on activities unnecessary to their families' wellbeing. In part field workers' sense of a pressure of time arose from their perceptions that rapid recruitment of large numbers of participants would reflect positively on their performance. In many instances, they met worried or even hostile responses from community members who were, as will be described later, suspicious of the unfamiliar heel prick sampling and the involvement of children who were healthy. Frustration was experienced by some field workers in relation to participants seen as 'difficult' if unable to understand information or reluctant to engage on the topic at all. Field workers found the information sheet used as part of the informed consent process limited in supporting communication about the research, due to its length and their perceptions of the messages being not particularly important.

Given these difficulties, field workers tended to use their own explanations in place of the information sheet. In doing this they often put more emphasis on SCD screening than genomics research, an approach they believed would help participants to understand the 'value of the research'. These issues were discussed during weekly feedback meetings, including the importance of the informed consent process and principles of voluntarism and informed choice. Counteracting beliefs that higher recruitment rates reflected more effective field worker performance appeared important in shifting attitudes and reducing a perceived pressure of time on home visits. Discussing recruitment challenges and positive experiences supported peer learning and underlined the inherent, as opposed to personal, nature of these difficulties. One such difficulty was balancing every person's right to refuse to participate with checking that refusals were not being made on the basis of simple misunderstandings that could be easily addressed.

A follow up training workshop was planned four months into recruitment to address these issues more comprehensively. This was an important opportunity to re-develop the study information and consent form, based on the experiences of the first months of communicating about this study. Using group work, the field workers developed a revised version in Kiswahili, drawing on their own communication skills, local knowledge and a structured tool to guide content. Their inclusion in this process reportedly increased their understanding of the study, and is likely to have strengthened their skills to explain it and increased their motivation to provide information in this agreed, standardised way; all of which potentially contribute to strengthening the informed consent process. The revised information sheet and consent form were subsequently submitted to and approved by the institutional and national ethics review committees.

### b) Consultations with chiefs

Before implementation, and following local and national science and ethics approval, members of the Community Liaison group explained and discussed the research with study area chiefs. This entailed providing information on the study, assessing chiefs' perceptions and attitudes towards it and asking for their recommendations, during a one day workshop conducted as part of a wider community engagement agenda. The findings are summarised below.

• Chiefs expressed general support for the research as a novel approach to vaccine and drug development in the future.

• Their main reservation was the acceptability of the heel prick; they expressed surprise at this method of taking blood and considered that people might not accept the procedure.

• Many chiefs expressed ideas indicating a good understanding of the heritability of physical disorders and illnesses, volunteering albinism as an example. These were described as being passed down through family lines, or clans (Kiswahili "*ukoo*"), through substances in the blood, blood cells or (for a few participants) in spermatozoa.

• Messages that centered on the concept of inherited resistance to disease, rather than on explanations about the way that transmission occurs through genes (or anything else), were thought to be most effective in communicating about the study. A range of approaches using farming analogies were presented and discussed, given the prominence of subsistence farming as a livelihood in this community. Chiefs identified a well known genetic predisposition and resistance to illness of certain breeds of goats and maize, reflected in their commercial value, as supportive of explanations for KGBC.

• SCD was not widely recognized either as an illness or a syndrome apart from by a few individuals with direct experience. The chiefs supported the inclusion of SCD screening as part of the research and suggested that carriers should also be identified and informed. Several expressed a view that SCD screening should be available to the public and encouraged for couples prior to marriage. Facilitators raised a number of ethical issues for genetic screening programmes and disclosure of carrier status, including the inability of children to give consent, the potential for stigmatization of carriers and misaligned paternity. The general consensus therefore supported the planned process to disclose SCD results to families affected by the disorder, and not to carriers.

• The export of samples to the UK for genotyping as part of this study and the archiving of blood samples for potential future research (contingent on local and national review and approval) were explained. No questions or discussion were generated by chiefs around these aspects of the research during this meeting or in subsequent informal conversations.

### c) Community meetings

Within the first six months of the start of this study, a series of 40 public meetings were organized by chiefs for community sensitization. Around 8000 people attended in total, with between 50 and 300 people per meeting. Messages on health research and KGBC were presented, alongside information on two other studies, and community facilitators addressed any questions raised. The same topics were later addressed in a series of 70 small group meetings with village elders, religious leaders and selected individuals seen as key opinion leaders. Reports of some religious leaders' negative attitudes to KEMRI's work led to their specific inclusion in this engagement activity. The main findings of these early community meetings are summarised below.

• Although attitudes were largely positive towards KEMRI, many questions reflected therapeutic 'misconceptions' of health research and consequent concerns about procedures, as has been reported for Kilifi elsewhere [11,24,28]

• The most frequently asked questions and strongest reaction, of surprise and concern, were about the heel prick procedure, as predicted by the chiefs. The heel prick was described as unfamiliar and therefore as raising fear in the community. One village elder wondered if his grandchild's death some time after a heel prick was associated with the procedure. Others raised concerns that it would 'make children unable to walk'. In one small group discussion, some mothers were said to 'hide their children when they see KEMRI field workers approaching their homes'. Importantly, the lack of familiarity with heel pricking as a procedure was also encountered amongst health providers, particularly nurses in peripheral health facilities, whose negative attitudes to the procedure at the outset of the study may have strengthened community concerns. Facilitators' explanations centred on safety and minimising discomfort, including the ways in which participants' rights and safety are protected in research.

• Clarification was often sought on the reasons that healthy rather than ill children would be recruited into the study. Facilitators' responses drew on descriptions of the differences between health research and medical treatment to explain that KGBC would involve healthy children because it did not have a primary individual therapeutic goal.

• There were many questions asked about the nature and signs of SCD, requiring facilitators to describe and explain this disorder. In small group meetings where more interaction was feasible, a component of the research that generated strongly positive comments was the return to each home of individual participants' results from the SCD screening test.

• Occasional questions were asked about samples being sent overseas. Responses to these based on the lack of availability of appropriate technology in Kenya were readily accepted.

• Although the archiving of samples for potential future research was described and protections outlined, no questions were raised about this procedure.

Many people arrived and left over the course of the large public meetings, and while some chose to ask questions, the understanding and reactions of the majority could not be assessed. Small group meetings were highly interactive and lively, generating many questions and sometimes extending discussions far beyond the areas anticipated. Overall, facilitators noted a positive change in participants' attitudes over the course of small group discussions. For example, a group that heatedly raised negative issues about KEMRI at the outset went on to include the welfare of KEMRI staff in closing prayers at the end of the meeting.

## Discussion

Over two years of a genetic cohort study in Kilifi, a series of activities have been conducted to support implementation as part of a wider systematic community engagement agenda, based on action research. This paper presents findings from an analysis of community engagement documentation and the experiences of the group closely involved in its implementation. There are potential methodological limitations, both for this form of document analysis and the influence of the authors, which are considered in the paragraphs below. Accepting these limitations, the findings presented here aim to illustrate the way in which the engagement activities conducted have enabled researchers to take account of community opinions and issues related to the study, and to build community understanding of research and greater levels of trust between researchers, field workers and community members. Key findings discussed include perceived challenges and facilitating factors in communicating about genetics/genomics research and KGBC; the critical role of field workers in supporting community understanding of research; and important challenges for consultation. We further explore theoretical and practical challenges in establishing the levels of consultation that are needed in specific research situations and put forwards suggestions for ways in which these difficulties might be addressed.

### Methodological limitations

Using documents as a source of data has methodological limitations for this analysis, including issues of authenticity, credibility, representativeness and meaning of the documents [49] as well as potential influence of the authors given their role in community engagement. In this research, the authenticity of the documents is not in doubt, but challenges could be raised for the other issues. Credibility and representativeness of the documents themselves have been strengthened by the attendance of an independent recorder, with training in qualitative data collection, at all community engagement activities with a stated role of documenting all proceedings rather than selecting any particular issues. The content may have been influenced by several factors, including the format of the meetings and the relationships between moderators and other participants. For example, it is possible that the community facilitators' affiliation with the research centre limited or influenced community expressions of dissatisfaction about KGBC, although the frequent voicing of negative opinions about the study suggests that the discussions were reasonably open. Further, the longstanding nature of the relationship between community facilitators and members may have been central to creating a sufficiently open atmosphere for discussion. Some support to the credibility and meaning of the content of reports is also provided by triangulation of many issues raised between different types of meetings (large and small groups) and participants (leaders, field workers and community members). A second set of limitations concerns the analysis of the documents, particularly given the roles of the authors in the activities covered by the reports. We have addressed this through following accepted methods for document analysis, acknowledging the need for reflexivity, involving more than one person in each step of the analysis and holding discussions with the wider community liaison group and between all authors to strengthen the trustworthiness of these findings.

### Communicating about genetics and genomics research

Although the literature points to challenges in communicating about genetics and genomics research, some aspects of KGBC were seen to facilitate community engagement in Kilifi and these may be common to other GWAS. Discussions on KGBC's goals were often facilitated by drawing on everyday observations and understandings. Although it is important to recognize the limitations of analogies in communication, facilitators were able to build their explanations on well known familial patterns of features, such as height, and agricultural knowledge and experience of resistance and susceptibility to disease [19]. Informed consent for research on a genetic condition in Ethiopia similarly drew successfully on general understandings of inheritance, and pointed to limitations in making detailed associations with inheritance patterns [50]. Other features that have supported community engagement in KGBC are that a discrete geographic community is involved, unlike some other forms of research or community, and that since healthy children are being recruited, there are opportunities for informed consent to be part of a process involving repeated discussions over time. Of particular importance is the ability to discuss the research with both parents of a potential participant through repeated visits.

The involvement of healthy children also brings particular challenges for communication, along with the large numbers of participants involved, the "one off" nature of the research interaction and the inclusion of SCD testing. In contrast to the widely known concepts referred to in the preceding paragraph, biomedical research is often not familiar and its conflation with the better known phenomenon of treatment leads to therapeutic 'misconceptions' [6,24]. For KGBC, the inclusion of SCD screening and the recruitment of healthy children seems, at least in some cases, to have generated a similar misinterpretation of research, but as a community-based health screening activity for individual-level benefit, or a 'health check'. Further, the policy of returning SCD results and the amount of information that field workers needed to provide on this disorder as part of the consent process are likely to have increased some participants' focus on SCD screening. These information challenges for field workers are exacerbated by the large number of participants and limited researcher-participant interactions involved in this type of research. The intention of strengthening participant benefits through SCD screening and the subsequent provision of medical services to affected children may therefore have paradoxically reduced awareness of the research goals of this study and undermined voluntary [32,33] informed consent for participation. The dilemma of whether or not to include SCD testing and disclosure of results in this study is increased by the wide community support voiced for this practice.

### The role of field workers in supporting community understanding of research

The findings highlight the key role that field workers play in supporting the ethics of informed consent [51]. Elsewhere [30,31], they have similarly been described as 'cultural brokers'. Field workers in KGBC are responsible for conveying the carefully developed and approved content of the information sheets and negotiating the informed consent process. They bring their own needs to this process of negotiation, as well as their understanding of the needs of the researchers and the community. At the same time, field workers often share the understandings and concerns of their neighbours and friends in the wider community [52]. They act as representatives of the institution, and their views on research, for example, are keenly listened to by others in the community. The findings show that interactions between the KGBC field workers and research participants have had an important impact on informed consent and therefore on the ethical conduct of this study in practice. These observations support the concept of a practical relations-based ethics in supplementing traditional principles-based paradigms [53,54]. In this respect, Gikonyo points out that current moves towards increasingly ambitious and stringent formal standards for information-giving to individuals should be counterbalanced with greater attention to the diverse social relationships that are essential to the successful application of these procedures.

Understanding the contribution that field workers make to a practical research ethics focuses attention on the need for long term and systematic capacity building. Training should be developed around recognition of the challenges that field workers face, and draw on behaviour change communication principles to address knowledge, attitudes and skills. We believe that facilitating field workers' development of the study information sheet, including piloting and translation into local languages, during a training workshop was a very important approach in this project. Where information and consent forms are submitted as part of an overall protocol review process well in advance of study implementation, as at KWTP, there may be logistical challenges in achieving this. However, amendments to information sheets arising from field worker training and monitoring processes early in the course of study implementation can be submitted for further approval, as was done here. Where these changes affect communication of content rather than content itself, re-submission to an ethics review board might only be required for information, rather than approval. An alternative that would avoid delays in implementing a final consenting process would be a two stage review process where consent forms are reviewed after the main protocol, closer to project implementation. In other situations, a separate formative research phase may be planned and it would be important to draw field workers for the main study into this preliminary process.

### Challenges for community consultation: What levels and methods should be used when?

Our experiences in KGBC illustrate that community consultation may focus mainly on issues that are directly experienced by the community and not raise issues on less visible costs or risks, unless these are deliberately raised and explored. The greatest initial concern about KGBC that arose from all sectors of the community concerned the heel prick. This was a very visible and unfamiliar procedure, entailing temporary discomfort for otherwise healthy children and therefore distress to parents. Reported concerns and rumours about the long term deleterious effects of this simple procedure were widespread at the outset, in contrast to the actual minimal potential harm. On the other hand, archiving of samples for future unspecified research (in this case, contingent on approval by local and national ethical review committees), a much less visible downstream research activity that attracts widespread international ethical debate and which was described as a procedure in this study, was not spontaneously identified as an issue through the consultation methods used in Kilifi. The importance of this finding is not likely to be that community members do not perceive these issues to be risks but that these were not recognised or considered. In a similar way, consultation has not provided insights into potential ethical implications of the policy on SCD screening in this study, excepting an early recognition that widespread population screening including carriers could generate issues over non-paternity. The activities planned did not aim to specifically explore community perceptions of risks around archiving or genetic screening but it is salient to note that the levels of consultation used here did not generate further exploration or debate of these issues. This raises questions about what level of consultation is appropriate to which research situations; how more intensive levels of consultation could be achieved, when needed; when this should be conducted in relation to implementation; and how the outcomes of consultation should in any case be taken into account. Dickert (2005) raises similar issues for community consultation; namely, identifying communities and stakeholders with legitimate interests, deciding when communities should be consulted, devising appropriate methods, knowing where these have been sufficient and incorporating community views into research plans [12].

### Was a greater depth of consultation needed for KGBC?

The literature suggests that an important feature determining the level of consultation needed is the risk involved in the research [14]. This model underlines the challenge for research where risks are difficult or impossible to predict, including those involving archiving for future undefined research. It could therefore be argued that, given that the protocol had already received science and ethics approval local, nationally and internationally, and that the potential costs or risks of archiving for future research are uncertain [23], seeking opinions and agreement on procedures including those concerning archive governance was an appropriate primary concern for consultation for KGBC. A converse view is that greater consultation on potential costs or risks in the study would have generated more autonomy at community, if not individual, level and potentially could have revealed ethical issues that did not emerge during the consultations conducted here. In addition, we cannot be certain how well the concepts of and safeguards for storing data for future use were understood in Kilifi and further community consultation on these systems is planned. However such debates are more appropriately related to general institutional policy than to study specific practice in situations where several different projects may include archiving practices over time, as at KWTP.

### How should more intensive consultation be done?

Where more intensive consultation is indicated, well recognised issues concern the way that this can be done, how findings of community consultation can be taken forwards and, connectedly, the timing of such activities. The most common model for consultation in biomedical research is the Community Advisory Board (CAB) [3]; alternative models include those from community participation in health policy [55-57] and experiences with deliberative forms of public debate in ethics [58-60]. Important methodological challenges for all these forms of community consultation include ensuring representativeness, particularly of members who have least influence, and accountability to the wider community [2-4,61,62]. Arising from these and other issues, there are challenges around taking the findings of community consultation into account in research policy and planning. One proposed approach that has been mentioned is that of deferring decision making to an independent review body, based on documentation of community consultation. This implies that consultation should be conducted at an early stage when a protocol can be adapted to incorporate recommendations. As discussed earlier for consent processes, separation of pilot and main phase protocol approval presents logistical challenges. Issues include funding and approval for the pilot phase itself and the consequences of perhaps repeatedly generating community expectations of research that does not go ahead, for reasons that may be unconnected with the consultation itself.

We propose that a potential source of extra support in addressing issues of representativeness and understanding how to take community perceptions and opinions into account in research planning could be derived from carefully designed social science studies feeding into empirical forms of ethical analysis [63-65]. Qualitative methods are uniquely able to explore such issues in depth and have strategies for addressing transferability of findings to wider or other populations. There is a need for more such research, in part to establish the support that this approach can provide to community consultation. Empirical ethics based on social science methods would supplement but clearly not replace the requirement for direct community engagement, given consultation goals of 'enhanced protection and benefits, legitimacy and shared responsibility' [12]. Direct community engagement therefore has an important procedural role, even where representativeness and accountability cannot be completely assured. We suggest that direct community engagement through wide researcher-community interactivity, sharing of documentation on community consultation with independent review bodies, and empirical ethics based on social science research can provide important, different and mutually supportive contributions to a process of community consultation, towards achieving its goals in practice.

## Conclusions

Community engagement in KGBC has provided opportunities for researchers to take account of community opinions and issues related to the study, and to build community understanding of research and greater levels of trust between researchers, field workers and community members.

The role of field workers was critical and their involvement in the development of materials fundamental in supporting informed consent processes. Discussions on concepts of inheritance were readily supported by drawing on existing knowledge and experience. This contrasted with efforts to create awareness and understanding of biomedical research. For KGBC, the more commonly described therapeutic 'misconception' of research tended to be translated into a perception of a community wide 'health check'. These challenges underline the need for systematic community engagement that draws on a range of methods, addresses different groups within the community and is sustained at least over the lifetime of the project. Community consultation was a particularly challenging concept to put into practice. Consultation can occur at different levels of intensity, and it is not straightforward to assess the complexity of information and levels of deliberation that are appropriate to a given study or the way that findings from such consultation can be taken into account. Drawing on these experiences and the literature on community engagement, we have considered some of the methods that could provide mutually supportive approaches to a process of community consultation in practice.

## Competing interests

The authors declare that they have no competing interests.

## Authors' contributions

VM was responsible for collating and analysing the data in this study and drafting the manuscript. DK and AM were responsible for managing the community engagement activities, supporting the collation and analysis of data and commenting on the manuscript. TW is the principal investigator for KGBC, supported the analysis plan and commented on the manuscript. SM supported the analysis and commented on the manuscript. All authors read and approved the final manuscript.

## Pre-publication history

The pre-publication history for this paper can be accessed here:

http://www.biomedcentral.com/1472-6939/11/13/prepub
